# ASNS-dependent asparagine availability supports intestinal ILC2 responses and type 2 immunity under nutrient-limited conditions

**DOI:** 10.3389/fimmu.2026.1878920

**Published:** 2026-09-15

**Authors:** Wenyu Tian, Xuesong Liu, Mengqi Zheng, Jing Shen

**Affiliations:** 1 Advanced Medical Research Institute, Shandong University, Jinan, China; 2 Department of Gastroenterology, Qilu Hospital of Shandong University, Jinan, China; 3 Suzhou Research Institute of Shandong University, Shandong University, Suzhou, Jiangsu, China

**Keywords:** anti-helminth immunity, asparagine, asparagine synthetase, group 2 innate lymphoid cell, immunometabolism

## Abstract

**Introduction:**

Nutrient availability critically governs group 2 innate lymphoid cell (ILC2) function and type 2 immunity. Here, we identify the non-essential amino acid asparagine (Asn) as a critical metabolite required for ILC2 survival and function.

**Methods:**

We investigated the roles of extracellular Asn availability and asparagine synthetase (ASNS)-mediated de novo Asn biosynthesis in ILC2 responses using complementary in vitro and in vivo approaches, including amino acid restriction, ILC2-specific *Asns* deletion, dietary Asn restriction, and *Nippostrongylus brasiliensis* infection.

**Results:**

ASNS, the rate-limiting enzyme for de novo Asn synthesis, was rapidly induced upon ILC2 activation under type 2 inflammatory conditions. When extracellular Asn was limited, ILC2s depended on ASNS-mediated Asn synthesis fueled by glutamine (Gln). Genetic ablation of *Asns* was well tolerated under Asnreplete conditions but severely impaired ILC2 viability and overall effector cytokine output upon Asn restriction. Dietary Asn restriction compromised ILC2s, and this effect was further aggravated by *Asns* deficiency. During *N. brasiliensis* infection, reduced intestinal Asn availability enhanced ILC2 reliance on Asns, whose deficiency impaired ILC2 abundance in the mLNs and protective anti-helminth immunity.

**Discussion:**

These findings identify Asn as a context-dependent metabolic adaptor that supports ILC2 viability and function under nutrient-limited and type 2 inflammatory conditions.

## Introduction

Group 2 innate lymphoid cells (ILC2s) are key effectors of type 2 immunity at barrier tissues and are particularly important in the intestine, where they contribute to mucosal homeostasis, tissue adaptation, and anti-helminth defense. Upon activation by epithelial-derived cytokines such as IL-33, IL-25, and thymic stromal lymphopoietin (TSLP), ILC2s rapidly produce type 2 cytokines including IL-5 and IL-13, thereby promoting eosinophil recruitment, mucus secretion, and helminth expulsion (1–3).

Metabolic reprogramming has emerged as a fundamental regulatory mechanism that supports ILC2 activation, expansion, tissue adaptation, and effector cytokine production (4–8). Upon stimulation by epithelial-derived cytokines, ILC2s rapidly increase their biosynthetic and energetic demands, which requires coordinated remodeling of nutrient uptake and intracellular metabolic pathways. Previous studies have shown that activated ILC2s undergo enhanced glycolytic activity, which supports rapid proliferation and type 2 cytokine production. In parallel, lipid metabolic programs also play important roles in ILC2 biology. Fatty acid uptake and oxidation contribute to ILC2-mediated barrier protection during malnutrition and helminth infection (5), whereas lipid-droplet formation and fatty acid handling have been linked to pathogenic ILC2 responses in allergic inflammation (6).

In addition to glycolysis and lipid metabolism, several nutrient-sensing and metabolic regulatory pathways have been identified as key modulators of ILC2 function. Arginase 1 acts as an ILC2-intrinsic metabolic checkpoint that regulates type 2 inflammation (4). mTORC1 signaling integrates nutrient availability and growth signals to support ILC2 proliferation and effector function (9), whereas LKB1/AMPK-related pathways are involved in maintaining metabolic fitness and cellular homeostasis (10). HIF-1α-associated programs have also been implicated in shaping ILC2 activation and inflammatory responses under metabolically demanding conditions (11). These findings indicate that ILC2s depend on multiple interconnected metabolic pathways to sustain their activation state and tissue-specific functions.

Amino acid uptake and availability represent another important layer of metabolic regulation in ILC2s. The amino acid transporter SLC7A8 has been shown to support ILC2 homeostasis and activation-dependent fitness, and amino acid availability has been proposed to act as a metabolic rheostat that determines the magnitude of ILC2 responses (7, 8). Notably, metabolomic analyses of activated lung ILC2s revealed relative enrichment of several amino acids, including L-alanine, valine, leucine, and isoleucine, highlighting the close relationship between amino acid metabolism and ILC2 activation. Among amino acid pathways, asparagine (Asn) is of particular interest because it can be obtained either through extracellular uptake or through *de novo* synthesis catalyzed by asparagine synthetase (ASNS) (12), which utilizes aspartate (Asp) as the carbon source and glutamine (Gln) as the amide nitrogen donor. Asn promotes CD8^+^ T cell activation by coordinating extracellular Asn uptake and ASNS expression and boosting LCK signaling, whereas Asn restriction improves CD8^+^ T cell metabolic fitness and antitumor function via an NRF2-dependent stress response (13, 14). Asn availability also governs germinal center B cell homeostasis (15). However, whether Asn availability and ASNS-dependent biosynthesis play comparable roles in ILC2 activation and function remains unknown.

In this study, we investigated whether Asn availability and ASNS-dependent *de novo* Asn biosynthesis supports ILC2 survival, effector cytokine production, and type 2 immune responses. We show that ILC2 activation is coupled to early induction of *Asns* expression, suggesting an adaptation of Asn biosynthesis in response to type 2 inflammatory contexts. Functionally, activated and expanded ILC2s require Asn availability to maintain survival and effector cytokine production under nutrient-limited conditions. Using genetic and dietary approaches, we further show that extracellular Asn availability and endogenous Asn synthesis contribute to intestinal ILC2 homeostasis *in vivo*. Finally, because *Nippostrongylus brasiliensis* infection represents a well-established intestinal type 2 inflammatory model characterized by robust ILC2 activation, we used this model to examine whether ASNS-dependent Asn biosynthesis is associated with ILC2 responses under inflammatory conditions *in vivo*. Together, these findings support a context-dependent role for Asn availability and ASNS-dependent Asn biosynthesis in sustaining intestinal ILC2 responses under nutrient-limited and type 2 inflammatory conditions.

## Materials and methods

### Mice

Male and female mice at the age of 6–8 weeks old were used for all animal experiments. C57BL/6 WT mice and *Asns*^fl/fl^ mice (background: C57BL/6J, strain T012991) were purchased from Gempharmatech (Nanjing, China). *Il5*^RFP-cre/+^ mice (strain 030926) was procured from The Jackson Laboratory. The *Asns*^fl/fl^ mice were crossed to *Il5*^RFP-cre/+^ mice to obtain a strain with *Asns*^fl/fl^ *Il5*^RFP-cre/+^ conditional gene deletion and *Asns*^+/+^ *Il5*^RFP-cre/+^ WT littermates (16–18). All the mice were housed in specific-pathogen-free facilities in ventilated cages with *ad libitum* food and water at the Model Animal Research Center of Shandong University.

### Public transcriptomic and metabolomic data analysis

Publicly available transcriptomic datasets were analyzed to evaluate the expression of Asn metabolism-related genes in ILC2s under different type 2 inflammatory conditions. GSE205669 contains bulk RNA-seq data from sorted small intestinal ILC2s stimulated with IL-25 for 48 h. E-MTAB-15989 contains bulk RNA-seq data from lung ILC2s sorted from papain-induced asthma model mice. GSE271362 contains bulk RNA-seq data from sorted small intestinal ILC2s stimulated with ADM2 for 3h. For these bulk RNA-seq datasets, raw FASTQ files were first subjected to quality control and adapter trimming using fastp (version 0.19.5). The filtered reads were then aligned to the mouse reference genome GRCm39 using STAR (version 2.7.9a). The resulting BAM files were processed with featureCounts (version 2.0.3) to generate raw gene count matrices, and differentially expressed genes were identified using DESeq2 (version 1.46.0).

GSE148539 contains Affymetrix Mouse Gene 2.0 ST Array data from lung ILC2s sorted from IL-33-treated mice. The expression matrix was generated using the mogene20sttranscriptcluster.db (version 8.8.0) and pd.mogene.2.0.st (version 3.14.1) annotation packages, and differential expression analysis was performed using limma (version 3.62.2).

All normalized gene expression matrix are provided as **Supplementary Materials** (**Supplementary Tables S1–S4**).

### Isolation of immune cells from intestinal lamina propria

The isolation of mouse immune cells from intestinal lamina propria was done as previously described (19). Briefly, intestines were separated and fat tissues were removed. Intestines were cut open and washed in cold PBS, and were then cut into 1 cm-long pieces. The tissues were then incubated in PBS containing 10 mM EDTA and 10 mM HEPEs with shaking 200 rpm at 37 °C for 30 min. The tissues were then digested in RPMI1640 containing FBS (5%), 1% penicillin-streptomycin, DNase I (150 U/mL, Sigma) and collagenase VIII (100 U/mL, Sigma) at 37 °C in a 5% CO_2_ incubator for 1.5 h. The digested tissues were shaken and filtered through 100 μm cell strainers. Mononuclear cells were then harvested from the interphase of an 80% and 40% Percoll (Cytiva) gradient after a spin at 2500 rpm for 15 min at room temperature. After density gradient centrifugation, the cells in the intermediate layer were collected, which were identified as lamina propria lymphocytes.

### Flow cytometry

For cytokine production, cells were stimulated *ex vivo* by 50 ng/mL PMA (PeproTech), 500 ng/mL Ionomycin (BioGems) for 4 h, and 2 μg/mL Brefeldin A (BioGems) was added 2 h before cells were harvested. Then cells were discriminated by Zombie Aqua Fixable Viability Kit (BioLegend) in PBS. Fc block (CD16/32) antibody was used to block the non-specific binding to Fc receptors before surface staining. Cells were stained with surface antibodies for 25 min at 4 °C. For intracellular staining, cells were incubated with Fixation/Permeabilization at 4 °C for 12 h. Then the cells were incubated with the intracellular antibodies for 2 h at 4 °C.

For flow cytometric analysis, lymphocytes were first identified by FSC-A and SSC-A, followed by exclusion of doublets and dead cells. ILC2s were defined as live CD45.2^+^Lin^−^Gata3^+^ or live CD45.2^+^Lin^−^KLRG1^+^ cells.

For cell sorting, large intestinal lamina propria ILC2s were sorted as live CD45.2^+^Lin^−^CD127^+^KLRG1^+^ cells. The lineage cocktail included CD3e, CD11b, CD11c, B220, CD19, CD5, Ter119, FcϵRIα, Ly6G and CD16/32.

All antibodies used in this study can be found in **Supplementary Table 2**.

### Cell culture

Sorted ILC2s were cultured and expanded in indicated IMDM media (Macgene CM10016), DMEM (Macgene CM15019), and DMEM(-Gln) (Macgene CM15013) supplemented with 15% FBS, mIL-2 (10 ng/mL, PeproTech), mIL-7 (10 ng/mL, PeproTech), mIL-25 (10 ng/mL, PeproTech) and mIL-33 (10 ng/mL, PeproTech).

For Asn/Asp rescue experiments, after expansion, cells were transferred into IMDM, DMEM, or DMEM(-Gln) for 48 h. For rescue experiments, Asn (28.4 mg/L) or Asp (30 mg/L) was added to the indicated media.

### Quantitative real-time RT-PCR

Sorted and cultured ILC2s were dissolved in RNA isolation reagent (Vazyme) and total RNA was isolated. cDNA was synthesized from extracted total-RNA using Reverse Transcriptase kit (Vazyme) according to the manufacturer’s protocol. Quantitative PCR was performed with SYBR-Green premix (Vazyme) and detected by a Real Time PCR System (StepOne, Applied Biosystems). The expression levels of target gene were normalized to the housekeeping gene *Gapdh*. 2^−ΔΔCt^ was used to calculate the relative mRNA expression of target genes. All primers used in this study are as follows:

*Gapdh* F: CATGGCCTCCAAGGAGTAAG, R: CCTAGGCCCCTCCTGTTATT;
*Asns* F: TCCAACCGGTCTTGTCACTG, R: AACACACAGCCAGAAACCCA;
*Il5* F: CTCTGTTGACAAGCAATGAGACG, R: TCTTCAGTATGTCTAGCCCCTG;
*Il13* F: CCTGGCTCTTGCTTGCCTT, R: GGTCTTGTGTGATGTTGCTCA;
*Gls* F: CATCCTCATCTGACGAGCGG, R: TCCTGTAGGATCTCCGAGGG;
*Gls2* F: CTTCCAAAAGTGTGTGAGCAGC, R: GGGATGTAGGCTGCCACTTT;
*Glul* F: GCAGGGAAACCCTAAGCAGT, R: GCAGGGAAACCCTAAGCAGT;
*Got1* F: GAAGACAATGGCTGACCGGA, R: TTTGGTGGCGTGAACTACGA;
*Slc1a4* F: CCTGGTGTTAGGAGTGGCTC, R: AGTCACTCTGGAACAGGTCG;
*Slc1a5* F: TGCCTTCCGCTCTTTTGCTA, R: GACGATAGCGAAGACCACCA;
*Slc7a1* F: ACTGTGGAAGGGCTCATTGT, R: CCGGCTAGGACATAGACACC;
*Slc7a5* F: CTGACACCTGTGCCATCACT, R: TTCACCTTGATGGGACGCTC;
*Slc7a8* F: AGGTGGAGGCGATCTGTTTC, R: GAGTGCGCTCCTACCCTCTA;
*Slc38a1* F: AGGGGCATAAGGTACACCGA, R: CGTGGAGCGGACCAGTTTAT;
*Slc38a2* F: GCCTGTTGTTGGATTACGGG, R: GGGATCCACGTCGGCATAAT.

### ELISA measurement of IL-5 and IL-13

IL-5 and IL-13 concentrations in cell culture supernatants were quantified using a Mouse IL-5 ELISA Kit (MULTI SCIENCES, Hangzhou, China) and a Mouse IL-13 ELISA Kit (MULTI SCIENCES, Hangzhou, China), respectively, according to the manufacturer’s instructions. Briefly, cell culture supernatants were collected and centrifuged at 1400 × g for 10 min to remove cell debris. The clarified supernatants were aliquoted and stored at -20 °C until analysis.

Before the assay, all reagents and samples were brought to room temperature. Standards were prepared by serial dilution to generate standard curves, and culture medium was used as the zero standard and dilution matrix for cell culture supernatant samples. For each assay, 100 μL of standards or cell culture supernatants was added to the pre-coated ELISA plate wells, followed by 50 μL of detection antibody working solution. The plate was sealed and incubated for 1.5 h at room temperature with gentle shaking. After washing six times with wash buffer, 100 μL of Streptavidin-HRP working solution was added to each well and incubated for 30 min at room temperature with gentle shaking. The plate was washed again, and 100 μL of TMB substrate was added to each well and incubated in the dark at room temperature for 5–30 min. The reaction was stopped by adding 100 μL of stop solution.

Absorbance was measured within 30 min using a microplate reader at 450 nm, with 630 nm used as the reference wavelength. Cytokine concentrations were calculated from the corresponding standard curves.

### Western blotting

ILC2s were lysed in RIPA lysis buffer containing protease inhibitors, and total protein concentrations were determined using a BCA protein assay kit. Equal amounts of protein were separated by SDS-PAGE and transferred onto PVDF membranes. After blocking with 5% non-fat milk at room temperature for 1 h, the membranes were incubated overnight at 4 °C with primary antibody against ASNS (Anti-Asparagine synthetase Rabbit pAb, Servicebio, 1:1000). After washing, the membranes were incubated with HRP-conjugated secondary antibody at room temperature for 1 h. ASNS protein bands were first detected at the expected molecular weight range of 55–64 kDa. After ASNS detection, the same membrane was washed and then incubated with anti-GAPDH antibody to detect the internal loading control. Protein bands were visualized using an enhanced chemiluminescence detection system and ASNS protein expression was normalized to GAPDH.

### Asn-deficient diet feeding

*Asns*^+/+^ *Il5*^RFP-cre/+^ and *Asns*^fl/fl^ *Il5*^RFP-cre/+^ mice were fed either a control diet (0.6% Asn, AIN76A, cat: A10021B, Jiangsu Synergy Pharmaceutical Bioengineering Co., Ltd.) or an Asn-deficient diet (0% Asn, based on AIN76A, Jiangsu Synergy Pharmaceutical Bioengineering Co., Ltd.) (20). All diets were isonitrogenous and contained similar calorie densities. All mice had *ad libitum* access to food and water. After three weeks, mice were sacrificed and intestinal tissues were collected.

### Measurements of tissue Asn

Intestinal Asn levels were quantified using an HPLC-MS system with an ADME column (100 mm × 2.1 mm). Approximately 30 mg SI or LI tissue was homogenized and extracted with 500 μL acetonitrile. Subsequently, 10 μL internal standard (IS) working solution (^13^C_10_^15^N_5_-GMP, 1 μg/ml, 10 μL) and 50 μL methanol–water (5:95, v/v) were sequentially added to the tissue lysate. The mixture was vortexed, followed by centrifuged at 12000 g for 20 min. A total of 400 μL supernatant was transferred to a new 1.5 mL tube and vacuum-dried to complete dryness at 30°C using a vacuum concentrator. The residue was reconstituted in 150 μL of initial mobile phase for instrumental detection.

Asn was detected in positive ion mode. The mobile phase contained solvent A (0.1% formic acid, FA) and solvent B (acetonitrile), with the gradient elution schedule detailed below: 0–3 min, 99% A; 3–4 min, 99–5% A; 4–6 min, 5% A; 6.1–8 min, 99% A. Respective standards were used to generate standard calibration curves. Samples were analyzed on a Qtrap 5500 (AB Sciex) system.

### *Nippostrongylus brasiliensis* infection model

For *Nippostrongylus brasiliensis* infection, L3 worms were obtained by the fecal culture method as described previously (21). Each mice received 500 L3 by s.c. injection. For intestinal worms’ recovery, small intestines were removed 5 days post-infection, placed in PBS and split longitudinally with sharp scissors. Then incubate samples for 2 h at 37 °C in a 50-mL centrifuge tube containing PBS, viable worms will collect at the bottom and total worms’ numbers were then counted using a dissecting microscope.

### Statistical analysis

Data are presented as the mean ± SD. Statistical analyses were performed using GraphPad Prism 10. For comparisons between two groups, the Mann-Whitney test was used. For comparisons among three or more groups, normality and homogeneity of variance were first assessed, and datasets included in parametric analyses met the assumptions of normal distribution and equal variance. Depending on the experimental design, ordinary one-way ANOVA followed by Tukey’s multiple comparisons test or ordinary one-way ANOVA followed by Dunnett’s multiple comparisons test was used. For datasets involving two independent factors, ordinary two-way ANOVA followed by Tukey’s multiple comparisons test was performed. A p value < 0.05 was considered statistically significant and is indicated as follows: *p < 0.05, **p < 0.01, ***p < 0.001, and ****p < 0.0001. Detailed statistical information for each experiment is provided in the corresponding figure legends.

## Results

### ILC2 activation is associated with induction of the Asn biosynthetic enzyme ASNS across type 2 inflammatory contexts

Asn derives from extracellular uptake or *Asns*-mediated biosynthesis from Asp and Gln (**Figure 1A**). To investigate how Asn metabolism is regulated during ILC2 activation, we isolated LI lamina propria ILC2s (**Supplementary Figure 1A**) and stimulated them *in vitro* with mIL-2, mIL-7, mIL-25 and mIL-33 (**Figure 1B**). Quantitative real-time PCR (qPCR) analysis showed that *Asns* expression was rapidly and robustly induced at an early stage after stimulation. Notably, the induction of *Asns* preceded that of canonical ILC2 effector genes (**Figure 1C**), suggesting that activation-associated induction of the Asn biosynthetic enzyme ASNS is an early transcriptional response accompanying ILC2 activation.

**Figure 1:**
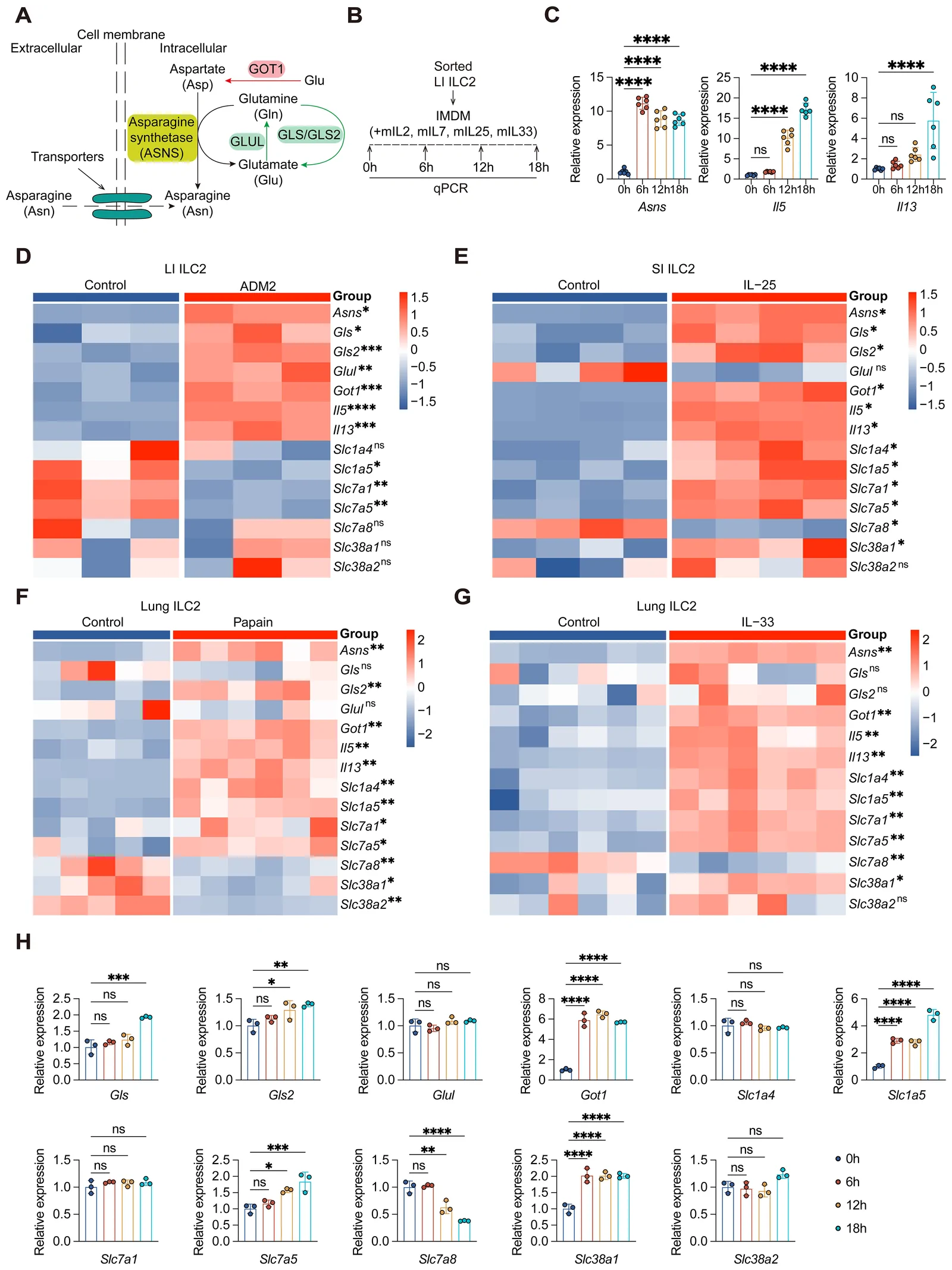
Asparagine synthetase is induced during ILC2 activation across inflammatory conditions. **(A)** Schematic illustration of asparagine (Asn) metabolism. **(B–C)** Large intestinal (LI) lamina propria ILC2s were sorted from C57BL/6 mice and then cultured in IMDM supplemented with mIL-2, mIL-7, mIL-25, and mIL-33 for the indicated durations, followed by qPCR analysis. Experimental workflow **(B)**. qPCR analysis of *Asns*, *Il5* and *Il13* expression in indicated time **(C)**. Each dot represents an individual mouse. **(D–G)** Heatmap showing the expression of the indicated genes in small intestinal (SI), LI or lung ILC2s under the indicated experimental conditions: ADM2 treatment *in vitro* (D, GSE271362), IL-25 stimulation *in vitro* (E, GSE205669), papain treatment (F, E-MTAB-15967), or IL-33 challenge (G, GSE148539). **(H)** qPCR analysis of selected Asn/Gln metabolism-related genes and amino acid transporter genes in LI ILC2s stimulated *in vitro* with IL-25/IL-33 for the indicated time points. Data are presented as mean ± SD. Statistical significance was determined by ordinary one-way ANOVA with Dunnett’s multiple comparisons test **(C, H)** or Mann-Whitney test **(D–G)**; ns, not significant; *p < 0.05; **p < 0.01; ***p < 0.001; ****p < 0.0001.

To determine whether activation-associated changes in Asn metabolism are observed across different type 2 inflammatory contexts, we analyzed publicly available transcriptomic datasets of ILC2s exposed to distinct stimuli or isolated from different tissues. We examined enzymes involved in Asn/Gln metabolism, as well as several SLC family transporters implicated in Asn/Gln uptake or exchange (22–26). Among these transporters, *Slc1a5* showed relatively high expression in small intestinal and lung ILC2s (**Supplementary Figure 1B**) and has been reported to function as an Asn transporter in T cells (24). We also performed GSEA to assess pathway-level changes in Asn/Asp/Gln metabolic programs (**Supplementary Figures 1C–F**).

In the publicly available datasets, *Asns* expression was increased in LI ILC2s following *in vitro* stimulation with adrenomedullin 2 (ADM2), together with increased expression of *Il5* and *Il13* (**Figure 1D**; GSE271362) (27). Likewise, mIL-25 stimulation of small intestinal (SI) ILC2s *in vitro* resulted in a marked increase in *Asns* expression, which coincided with the induction of *Il5* and *Il13* (**Figure 1E**; GSE205669) (28). In lung ILC2s, papain treatment or IL-33 stimulation was likewise associated with increased *Asns* expression and induction of *Il5* and *Il13* (**Figures 1F, G**; ArrayExpress: E-MTAB-15989; GSE148539) (29, 30). These independent datasets provide supportive evidence that increased *Asns* expression is associated with ILC2 activation across diverse type 2 inflammatory contexts, although the specific transcriptional responses may differ depending on the tissue and stimulus.

In addition to *Asns*, we further examined key enzymes involved in Asn/Gln metabolism that provide metabolic substrates for ASNS-mediated *de novo* Asn biosynthesis (**Figures 1D–F**). Notably, *Got1* was consistently upregulated across all tissue and stimulation conditions examined, suggesting an enhanced capacity for the conversion of Glu to Asp (**Figures 1D–F**). In addition, at least one of the Gln metabolism-associated genes, *Gls*, *Gls2*, or *Glul*, was upregulated in ILC2s under all examined conditions except IL-33-treated lung ILC2s, indicating that Gln metabolism is also transcriptionally enhanced across multiple ILC2 activation contexts (**Figures 1D–H**). These observations prompted us to determine whether similar metabolic changes occur under our experimental conditions. Under our IL-25/IL-33 stimulation conditions, *Got1*, *Gls* and *Gls2* were upregulated following activation (**Figure 1H**). Notably, the induction of *Got1* preceded that of the effector cytokine genes *Il5* and *Il13* (**Figures 1C, H**), suggesting that changes in Asn/Gln metabolism may represent an early transcriptional response during ILC2 activation. Together, these observations suggest that activation-associated changes in Asn/Gln metabolism may support the metabolic demands associated with *de novo* Asn biosynthesis.

The expression changes of Asn/Gln transporters appeared more complex and did not exhibit a uniform activation-associated signature (**Figures 1D–G**). ILC2 activation was accompanied by tissue- and stimulus-dependent remodeling of Asn/Gln transporter expression. Notably, several Slc genes, including *Slc1a4*, *Slc7a1*, *Slc7a5* and *Slc7a8* showed similar changes in activated SI and lung ILC2s (**Figures 1E–G**). Our analysis under IL-25/IL-33 stimulation further revealed increased expression of *Slc1a5*, *Slc7a5*, and *Slc38a1* in LI ILC2s (**Figure 1H**), further supporting activation-associated changes in amino acid transport under our experimental conditions. Notably, the induction of *Slc1a5* and *Slc38a1* also preceded the induction of *Il5* and *Il13* (**Figures 1C, H**), suggesting that changes in amino acid transport also occur early during ILC2 activation.

Across the publicly available datasets, GSEA revealed significant enrichment and upregulation of pathways associated with Asp and Gln catabolism/metabolism in activated ILC2s, further supporting the involvement of broader amino acid metabolic remodeling during ILC2 activation (**Supplementary Figures 1C–F**).

Collectively, these results indicate that *Asns* is induced at an early stage during IL-25/IL-33-stimulated LI ILC2 activation. Public transcriptomic datasets further support activation-associated *Asns* induction across multiple type 2 inflammatory contexts, whereas changes in Slc transporters and Asn/Gln metabolism-related genes appear to be tissue- and stimulus-dependent. Importantly, qPCR analysis under our IL-25/IL-33 stimulation conditions further confirmed demonstrated induction of key Asn/Gln metabolism-related genes and transporters, with *Got1*, *Slc1a5*, and *Slc38a1* showing induction before the effector cytokine genes *Il5* and *Il13*. These findings prompted us to investigate whether Asn availability contributes to ILC2 viability and effector cytokine production.

### Asn deprivation renders ILC2s dependent on *de novo* Asn synthesis

To define the role of Asn in ILC2 biology, we examined the effects of Asn and its biosynthetic substrates, Asp and Gln, under nutrition-limited conditions. LI lamina propria ILC2s were initially first activated and expanded under Asn-, Asp-, and Gln-replete conditions (IMDM) and then cultured in media containing either all nutrients (IMDM), lacking Asn and Asp (DMEM), or lacking Asn, Asp, and Gln (DMEM(-Gln)) (**Figures 2A, B**; **Supplementary Figure 2A**; **Supplementary Table 5**). We selected LI lamina propria ILC2s for the *in vitro* culture system because the LI represents a nutrient- and microbiota-exposed mucosal site where ILC2s are likely to encounter fluctuations in amino acid availability. In addition, LI ILC2s could be reproducibly sorted and expanded in sufficient numbers for amino acid-restriction and rescue assays.

**Figure 2:**
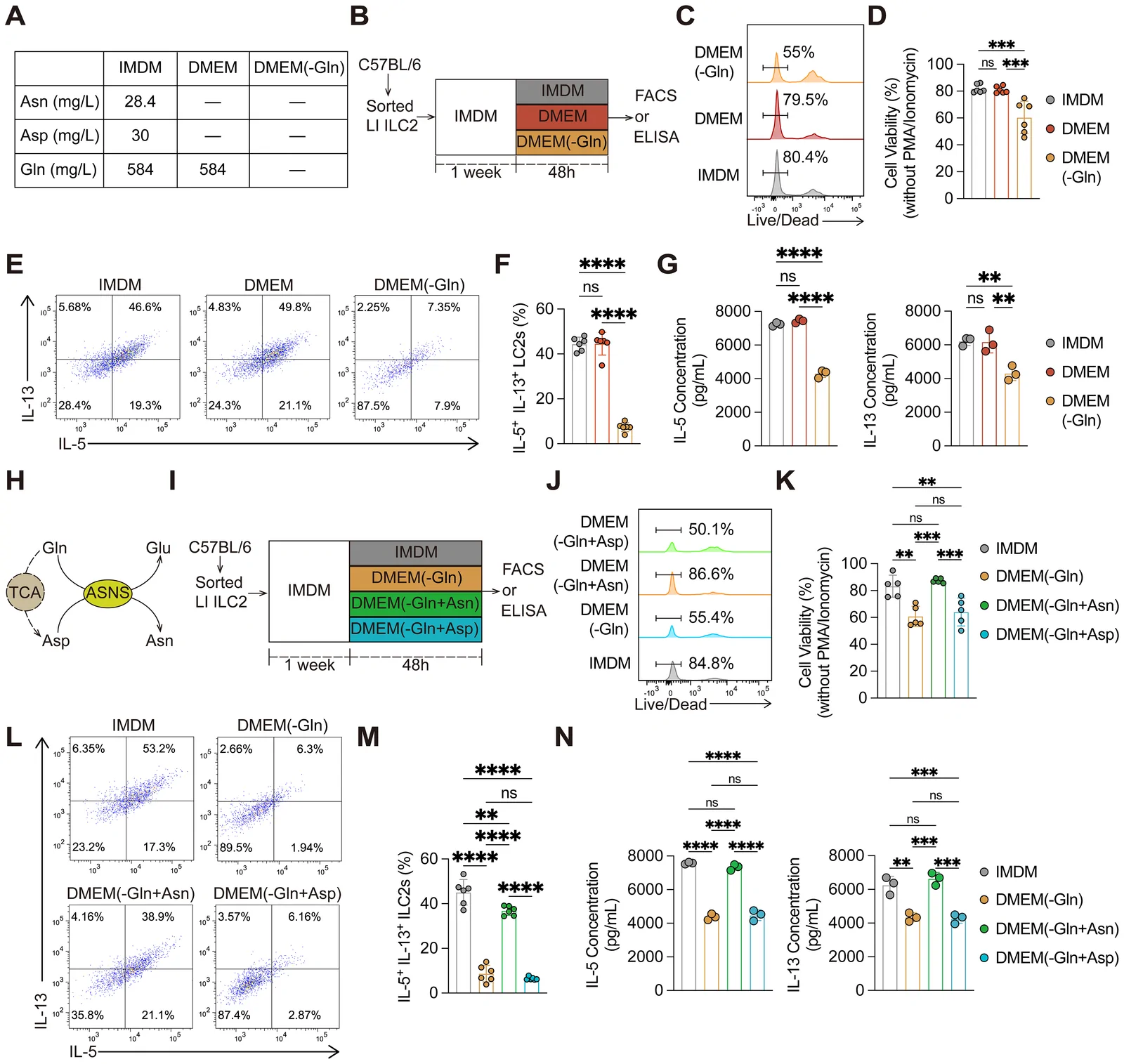
Asn deprivation renders ILC2s dependent on *de novo* Asn synthesis. **(A)** The concentrations of L-Asn, L-aspartate (Asp), and L-glutamine (Gln) in IMDM, DMEM, and glutamine-deficient DMEM (DMEM(-Gln)) medium. **(B–G)** LI lamina propria ILC2s were sorted from C57BL/6 mice, expanded in IMDM for 1 week, and then cultured for 48 h in IMDM, DMEM, or DMEM(-Gln), followed by flow cytometric analysis or ELISA. Experimental workflow **(B)**. Representative histograms showing the viability of ILC2s **(C)**. Quantification of the frequency of live cells (without PAM/Ionomycin/Brefeldin A stimulation) **(D)**. Representative flow cytometry plots showing IL-5 and IL-13 production by ILC2s **(E)**. Quantification of the frequencies of IL-5^+^ IL-13^+^ ILC2s **(F)**. Concentrations of IL-5 and IL-13 in ILC2 culture supernatants measured by ELISA **(G)**. Each dot represents an individual mouse. **(H)** Schematic illustration of the metabolic pathway linking Gln, Glu, Asp, and Asn. **(I–N)** LI lamina propria ILC2s were sorted from C57BL/6 mice, expanded in IMDM for 1 week, and then cultured for 48 h in IMDM, DMEM(-Gln) and DMEM(-Gln) supplemented with Asn (28.4 mg/L, DMEM(-Gln+Asn)) or Asp (30 mg/L, DMEM(-Gln+Asp)), followed by flow cytometric analysis or ELISA. Experimental workflow **(I)**. Representative histograms showing the viability of ILC2s **(J)**. Quantification of the frequency of live cells (without PAM/Ionomycin/Brefeldin A stimulation) **(K)**. Representative flow cytometry plots showing IL-5 and IL-13 production by ILC2s **(L)**. Quantification of the frequencies of IL-5^+^ IL-13^+^ ILC2s **(M)**. Concentrations of IL-5 and IL-13 in ILC2 culture supernatants measured by ELISA **(N)**. Each dot represents an individual mouse. Data are presented as mean ± SD. Statistical significance was determined by ordinary one-way ANOVA with Tukey’s multiple comparisons test; ns, not significant; **p < 0.01; ***p < 0.001; ****p < 0.0001.

Under DMEM conditions, in which Asn and Asp are absent but Gln is present, ILC2 viability and cytokine production were only minimally affected. In contrast, under DMEM(-Gln) conditions, in which Asn, Asp, and Gln are all absent, ILC2 viability was reduced (**Figures 2C, D**; **Supplementary Figures 2B, C**), accompanied by decreased IL-5 and IL-13 production measured by ELISA and flow cytometry (**Figures 2E–G**; **Supplementary Figures 2D, E**). These results suggest that Gln supports intracellular Asn biosynthesis not only by serving as an amide nitrogen donor but also by fueling metabolic pathways that maintain Asp production (**Figure 2H**).

Given that IMDM and DMEM differ not only in Asn, Asp, and Gln content but also in other amino acids, vitamins, and buffering systems, the direct comparison between these media was interpreted with caution. Therefore, to minimize potential confounding effects from differences in basal medium composition, we performed rescue experiments within the same DMEM(-Gln) background by supplementing either Asn or Asp. Asn supplementation but not Asp restored ILC2 viability and substantially rescued IL-5 and IL-13 production measured by ELISA and flow cytometry (**Figures 2I–N**; **Supplementary Figures 2F–I**). Although Asn supplementation improved intracellular IL-5 and IL-13 levels detected by flow cytometry, the rescue was incomplete (**Figures 2L, M**), which may reflect the additional metabolic stress imposed by PMA/ionomycin restimulation and Brefeldin A treatment during intracellular cytokine staining. These procedures require rapid cytokine synthesis and accumulation, thereby increasing biosynthetic demands that may not be fully supported in the absence of Gln. These results suggest that Gln supports ILC2 function, at least in part, by fueling *de novo* Asn biosynthesis. The incomplete rescue by exogenous Asn further suggests that Gln supports ILC2 responses through additional metabolic pathways beyond Asn biosynthesis.

In mammalian cells, including T and B lymphocytes, Asn restriction elicits a time-dependent upregulation of ASNS (13–15). In line with these observations, ILC2s similarly upregulated *Asns* expression in response to Asn deprivation (Supplementary **Figures 3A, B**).

**Figure 3:**
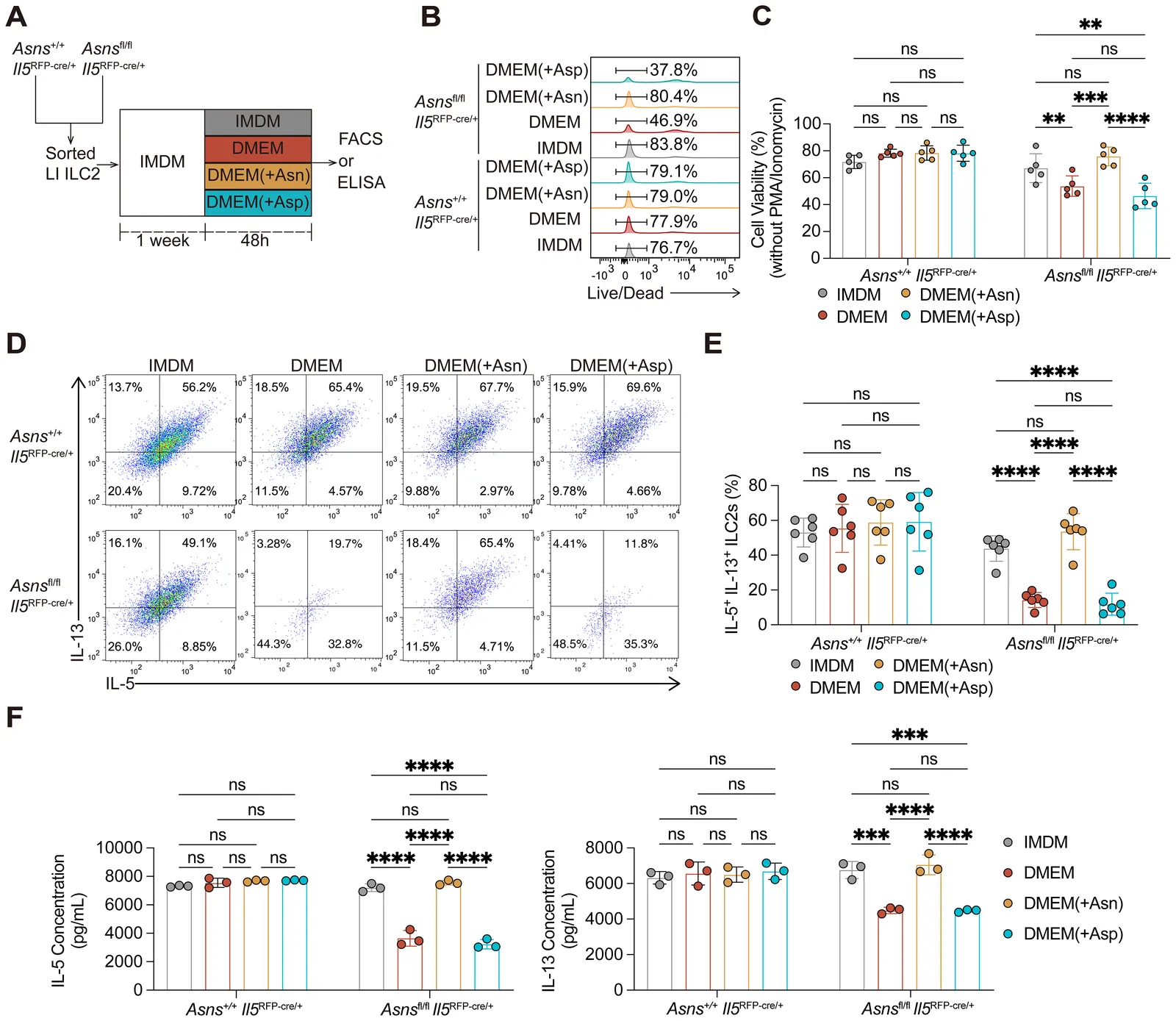
Glutamine deprivation impairs ILC2 function, which is partially rescued by exogenous asparagine. **(A–F)** LI lamina propria ILC2s were sorted from *Asns*^+/+^ *Il5*^RFP-cre/+^ (control) or *Asns*^fl/fl^ *Il5*^RFP-cre/+^ (ILC2-specific *Asns*-deficient) mice and expanded in IMDM for 1 week. Cells were then cultured for 48 h in IMDM, DMEM and DMEM supplemented with Asn (28.4 mg/L, DMEM(+Asn)) or Asp (30 mg/L, DMEM(+Asp)), followed by flow cytometric analysis or ELISA. Experimental workflow **(A)**. Representative histograms showing the viability of ILC2s **(B)**. Quantification of the frequency of live cells (without PAM/Ionomycin/Brefeldin A stimulation) **(C)**. Representative flow cytometry plots showing IL-5 and IL-13 production **(D)**. Quantification of the frequencies of IL-5^+^ IL-13^+^ ILC2s **(E)**. Concentrations of IL-5 and IL-13 in ILC2 culture supernatants measured by ELISA **(F)**. Each dot represents an individual mouse. Data are presented as mean ± SD. Statistical significance was determined by ordinary two-way ANOVA with Tukey’s multiple comparisons test; ns, not significant; **p < 0.01; ***p < 0.001; ****p < 0.0001.

In summary, these results indicate that, in this *in vitro* culture system, ILC2s that had been activated and expanded in culture with IL-2, IL-7, IL-25, and IL-33 require Asn availability to maintain cell survival and effector cytokine production. Under Asn-restricted conditions, Gln may support ILC2 function at least in part by fueling ASNS-dependent *de novo* Asn biosynthesis, while additional Gln-dependent metabolic pathways may also contribute to the maintenance of ILC2 activity.

### ASNS sustains ILC2 survival and function under extracellular Asn limitation

Based on this observation, we next asked whether ASNS-mediated *de novo* Asn synthesis contributes to ILC2 when extracellular Asn is limited. Although *Asns* is upregulated during ILC2 activation (**Figures 1C–G**) and in response to extracellular Asn deprivation (**Supplementary Figures 3A, B**), the functional contribution of ASNS to ILC2s under Asn-limited conditions remained unclear. To address this, we generated an ILC2-specific *Asns*-deficient (*Asns*^fl/fl^ *Il5*^RFP-cre/+^) mouse strain by crossing *Asns*^fl/fl^ mice with *Il5*^RFP-cre/+^ mice (16–18) (**Supplementary Figures 3C, D**). LI lamina propria ILC2s were isolated from *Asns*-deficient mice and their littermate controls, and expanded in IMDM. Under Asn, Asp, and Gln-replete conditions (IMDM), *Asns* deletion did not affect ILC2 viability or cytokine production (**Supplementary Figures 3E–I**), indicating that extracellular Asn can support ILC2 survival and effector cytokine production in the absence of endogenous Asn synthesis.

To examine the contribution of *Asns* when extracellular Asn is limited, control and *Asns*-deficient ILC2s were cultured in either Asn-sufficient IMDM or Asn-deficient DMEM following expansion (**Figure 3A**). Under Asn-deficient conditions (DMEM), *Asns*-deficient ILC2s exhibited decreased cell viability together with reduced IL-5 and IL-13 production measured by flow cytometry and ELISA compared with control ILC2s (**Figures 3B–F**; **Supplementary Figures 4A–D**). These findings suggest that ASNS-mediated *de novo* Asn synthesis contributes to maintaining ILC2 viability and effector cytokine production when extracellular Asn availability is limited. These results also showed that even under Asn-deprived, Gln-supplemented conditions, *Asns* deficiency was associated with reduced ILC2 viability and effector cytokine function. These findings suggest that the ability of Gln to support ILC2 function under Asn-limited conditions may depend, at least in part, on ASNS-mediated Asn biosynthesis.

To confirm that the functional defects of *Asns*-deficient ILC2s in DMEM were specifically due to impaired Asn biosynthesis, we performed rescue experiments by supplementing DMEM with either Asn or Asp (**Figure 3A**). In *Asns*-deficient ILC2s, exogenous Asn but not Asp substantially rescued cell viability, and IL-5, IL-13 production as measured by flow cytometry and ELISA (**Figures 3B–F**; **Supplementary Figures 4B–D**). qPCR analysis showed a similar but less pronounced reduction in *Il5* and *Il13* mRNA expression compared with intracellular cytokine production (**Supplementary Figures 4E, F**). As a substrate for protein synthesis, Asn availability can influence translational capacity, particularly in highly activated cells with increased demands for *de novo* protein production (7, 8, 31, 32). Thus, although cytokine gene transcription may be partially maintained under Asn-restricted conditions, limited Asn availability may constrain efficient protein synthesis and cytokine accumulation, resulting in a greater reduction in cytokine production than in cytokine mRNA expression.

Collectively, these findings suggest that extracellular Asn uptake and ASNS-mediated Asn *de novo* synthesis both contribute to the maintenance of ILC2 viability and effector cytokine production.

### ASNS-mediated Asn biosynthesis maintains intestinal ILC2 homeostasis under Asn-limited conditions *in vivo*

We next investigated whether environmental Asn availability regulates ILC2 responses *in vivo*. Under steady-state conditions, *Asns* deficiency did not affect the frequency of intestinal ILC2s or the proportion of RFP^+^ cells, a reporter reflecting *Il5* transcription (**Supplementary Figure 5A**, **Figures 4A–H**), suggesting that exogenous Asn derived from the diet is sufficient to sustain ILC2 homeostasis when endogenous synthesis is absent.

**Figure 4:**
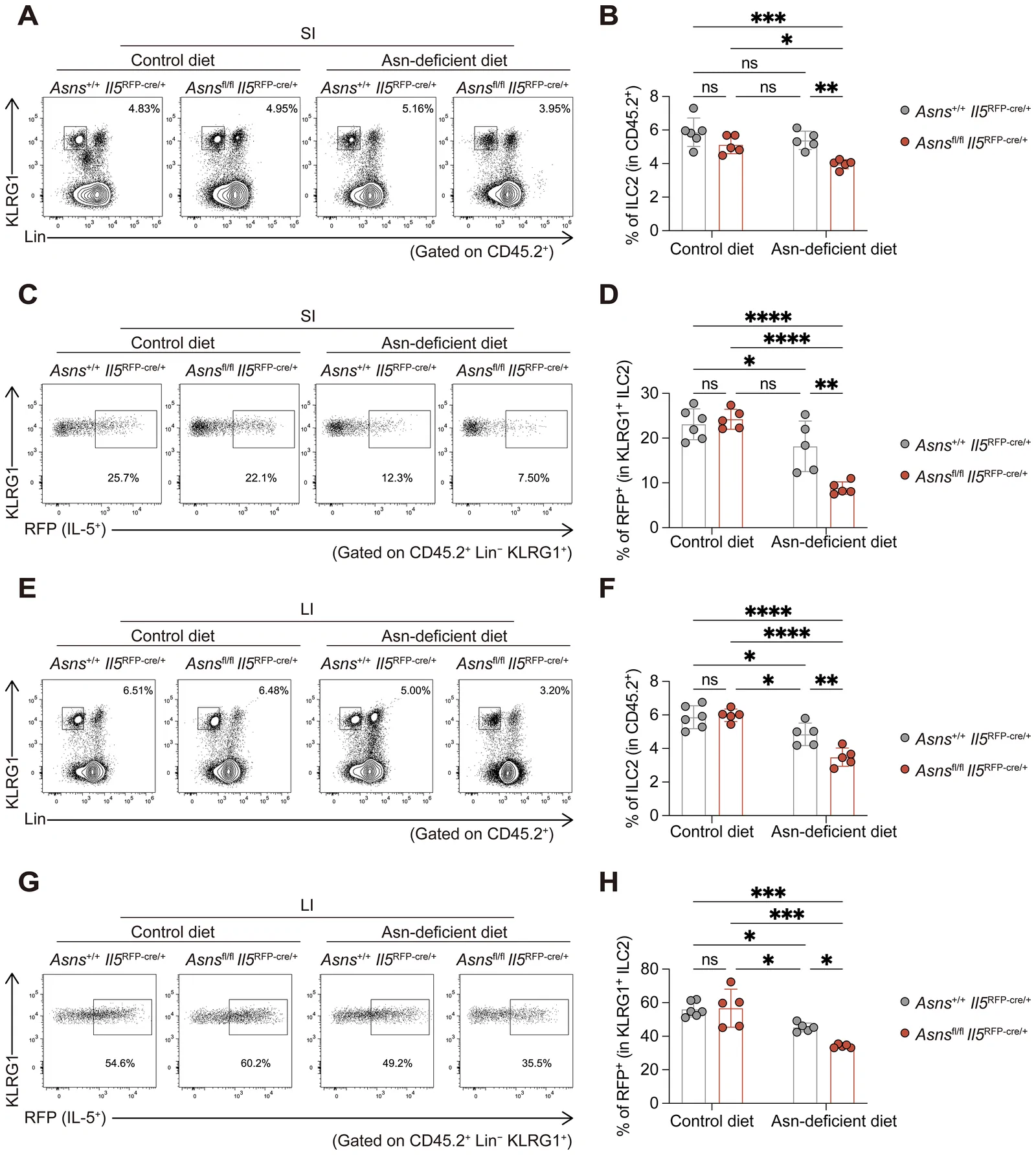
Environmental Asn supports intestinal ILC2 homeostasis *in vivo.* **(A–H)** *Asns*^+/+^ *Il5*^RFP-cre/+^ and *Asns*^fl/fl^ *Il5*^RFP-cre/+^ (ILC2-specific *Asns*-deficient) mice were fed either an Asn-deficient diet or a control diet for three weeks. Representative flow cytometry plots **(A, E)** and quantification **(B, F)** of KLRG1^+^ ILC2s in the SI **(B)** and LI **(F)** lamina propria lymphoid. Representative flow cytometry plots **(C, G)** and quantification **(D, H)** of RFP^+^ ILC2 frequencies within CD45.2^+^ Lin^–^ KLRG1^+^ ILC2s in the SI **(D)** and LI **(H)** lamina propria lymphoid. Each dot represents an individual mouse. Data are presented as mean ± SD. Statistical significance was determined by ordinary two-way ANOVA with Tukey’s multiple comparisons test; ns, not significant; *p < 0.05; **p < 0.01; ***p < 0.001; ****p < 0.0001.

To directly test the contribution of environmental Asn, *Asns*^fl/fl^ *Il5*^RFP-cre/+^ mice and their littermate controls were fed either an Asn-deficient or a control diet for three weeks to reduce systemic Asn levels and limit its availability to intestinal ILC2s (**Supplementary Figure 5B**). Previous studies have also demonstrated that this Asn-deficient dietary regimen effectively lowers circulating Asn levels in mice (20). In control mice, dietary Asn deprivation reduced the frequency of ILC2s in the large intestine and decreased the proportion of RFP^+^ ILC2s in both the small intestine and large intestine (**Figures 4A–H**). These findings indicate that prolonged reduction in environmental Asn availability is associated with impaired intestinal ILC2 homeostasis under physiological conditions.

Notably, under an Asn-deficient conditions, both the frequencies of ILC2s and RFP^+^ ILC2s of in the colon and small intestine were further diminished in *Asns*^fl/fl^*Il5*^RFP-cre/+^ mice compared with control mice (**Figures 4A–H**; **Supplementary Figure 5C**). Together, these findings suggest that environmental Asn availability and ASNS-dependent endogenous Asn synthesis both contribute to maintaining intestinal ILC2 homeostasis, particularly under conditions of prolonged Asn availability.

### ASNS deficiency is associated with reduced mLN ILC2 accumulation and increased worm burden during *N. brasiliensis* infection

Having shown that dietary Asn restriction compromises intestinal ILC2 homeostasis, we next asked whether ASNS-dependent Asn biosynthesis is associated with anti-helminth immunity in a physiologically relevant intestinal type 2 inflammatory setting. We therefore used *N. brasiliensi*s infection, a well-established helminth-induced type 2 inflammation model. To assess whether Asn availability is altered in the intestinal environment during infection, we analyzed publicly available metabolomic data from small intestinal contents. Notably, Asn levels were significantly decreased in *N. brasiliensis*–infected mice compared with control mice, suggesting reduced Asn availability in the intestinal microenvironment during *N. brasiliensis* infection (**Figure 5A**, MTBLS3486) (33).

**Figure 5:**
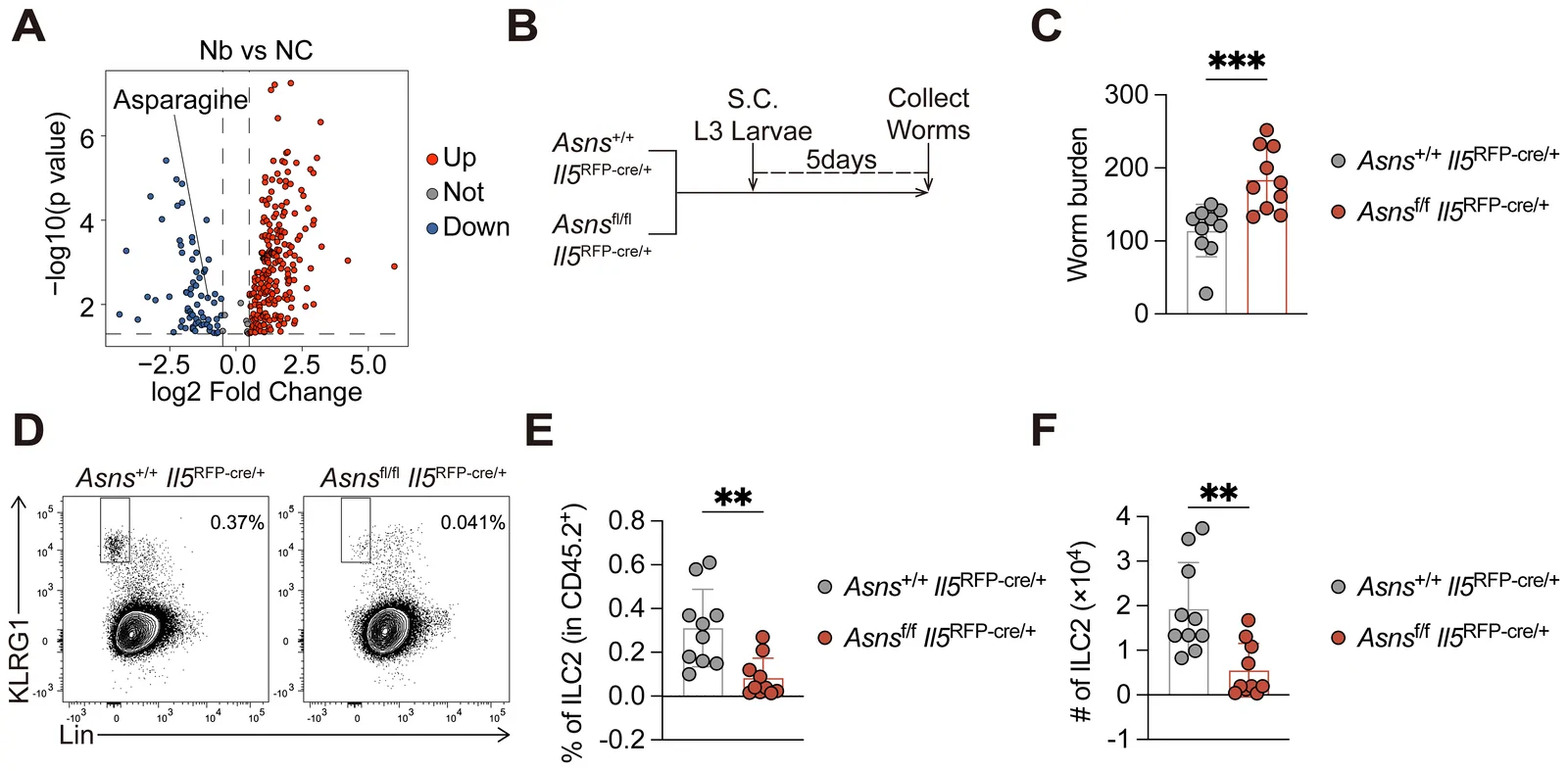
ASNS deficiency is associated with reduced mLN ILC2 abundance and increased worm burden during *N. brasiliensis* infection. **(A)** Volcano plot showing differential metabolites in small intestinal contents from *N. brasiliensis*-infected mice (Nb) compared with Negative control mice (NC) (MTBLS3486). Each dot represents a metabolite. Red, upregulated; blue, downregulated; gray, not significant. **(B–F)** *Asns*^fl/fl^*Il5*^RFP-cre/+^ mice and their littermate controls were inoculated subcutaneously with *N. brasiliensis* L3 larvae for 5 days. Experimental workflow **(B)**. Quantification of intestinal worm burden at day 5 post-infection (n=10) **(C)**. Representative flow cytometry plots **(D)**, quantification of KLRG1^+^ ILC2 frequency **(E)** and quantification of total number of KLRG1^+^ ILC2 **(F)** in mesenteric lymph node at day 5 post-infection (n=10). Each dot represents an individual mouse. Data are presented as mean ± SD. Statistical significance was determined by Mann-Whitney test; **p < 0.01; ***p < 0.001.

To determine whether ILC2-specific *Asns* deficiency affects host resistance to N. brasiliensis infection, *Asns*^fl/fl^ *Il5*^RFP-cre/+^ mice and their littermate controls were inoculated subcutaneously with *N. brasiliensis* L3 larvae, and intestinal worm burden was assessed 5 days post-infection (**Figure 5B**). Notably, *Asns*-deficient mice exhibited a significantly higher intestinal worm burden than control mice, suggesting reduced host resistance to *N. brasiliensis* infection (**Figure 5C**).

We quantified ILC2s in the mesenteric lymph nodes (mLNs), an important site of immune cell accumulation during helminth infection. Flow cytometric analysis revealed a marked reduction in both the frequency and the total number of ILC2s in the mLNs of infected *Asns*-deficient mice compared with controls (**Figures 5D–F**). These findings indicate that mLN ILC2 abundance is reduced in *Asns*-deficient mice during *N. brasiliensis* infection.

Together, these findings suggest that reduced environmental Asn availability during infection and impaired ASNS-dependent Asn biosynthesis in ILC2s are associated with increased intestinal worm burden. The reduced frequency and the total number of mLN ILC2s provides an indirect indication of an altered intestinal-associated type 2 immune response in *Asns*-deficient mice. However, whether the altered mLN ILC2 response reflects changes in environmental Asn availability, cell-intrinsic effects of *Asns* deficiency, or a combination of both remains to be determined.

## Discussion

During type 2 inflammation, activated immune cells, epithelial cells, and tissue remodeling substantially increase local metabolic demand, which may alter amino acid availability within inflamed tissues. Consistent with this possibility, we observed reduced intestinal asparagine levels following *N. brasiliensis* infection together with induction of *Asns* expression in activated ILC2s. These findings suggest that *de novo* Asn biosynthesis may represent an adaptive mechanism that enables ILC2s to maintain effector function when extracellular Asn becomes limiting during inflammation. However, the mechanisms responsible for local Asn depletion and the broader physiological settings in which Asn restriction occurs remain to be investigated.

*Asns* supports ILC2 responses during helminth infection and may contribute to effective parasite expulsion, although potential contributions from Th2 cells and other IL-5-expressing immune cells cannot be fully excluded. ILC2s and Th2 cells both produce type 2 cytokines such as IL-5 and IL-13, but they differ in their activation kinetics and upstream regulatory mechanisms. ILC2s rapidly respond to epithelial-derived cytokines, including IL-25, IL-33, and TSLP, and are important early contributors to type 2 immunity, whereas Th2 cells require antigen recognition, clonal expansion, and differentiation and are generally more involved in amplifying and sustaining adaptive type 2 immune responses. In the present study, worm burden and ILC2 responses were assessed at day 5 after *N. brasiliensis* infection, a time point at which early innate type 2 responses are highly relevant. Nevertheless, because the *Il5*^RFP-cre/+^ model may target multiple IL-5-expressing cell types, including Th2 cells under inflammatory conditions, we cannot fully exclude the contribution of Th2 cells or other IL-5-expressing immune cells to the *in vivo* phenotype. Therefore, the impaired parasite expulsion observed in *Asns^fl^*^/fl^ *Il5*^RFP-cre/+^ mice should be interpreted as resulting from *Asns* deficiency in IL-5-expressing type 2 immune cells, accompanied by defective ILC2 responses. Future studies using more selective ILC2-targeting models, Th2 cell analysis, Rag-deficient mice, or ILC2 adoptive transfer approaches will be needed to distinguish ILC2-intrinsic effects from Th2-dependent mechanisms.

Our *in vitro* amino acid-restriction experiments were performed after ILC2s had been activated and expanded in cytokine-containing IMDM. Therefore, these assays primarily assess how Asn/Gln availability sustains the viability and effector function of activated ILC2s. Whether Asn availability is required during the initial activation of naïve ILC2s will require additional experiments in which freshly isolated ILC2s are stimulated under defined amino acid conditions. An additional consideration is that Asn/Gln restriction substantially affected ILC2 viability, which may confound the interpretation of IL-5 and IL-13 production. Therefore, the reduction in IL-5^+^ and IL-13^+^ ILC2s under amino acid-restricted conditions should not be interpreted solely as a direct suppression of cytokine expression. Rather, our data indicate that Asn/Gln availability supports the overall effector output of activated/expanded ILC2s, at least in part by maintaining cell survival.

Because IL-25 and IL-33 may activate partially distinct ILC2 programs and could differentially influence cellular metabolism, our combined cytokine culture system does not distinguish cytokine-specific metabolic effects. Future experiments comparing IL-25 alone, IL-33 alone, and combined IL-25/IL-33 stimulation will be important to determine whether *Asns* induction and Asn dependence are regulated in a cytokine-specific manner.

Gln, the most abundant amino acid in human serum, is essential for the proliferation and survival of most mammalian cells *in vitro* (34, 35). Notably, the absence of exogenous Gln can be functionally compensated by Asn supplementation, which restores cellular growth and protein synthesis (36, 37). Consistent with this concept, we find that Gln deprivation does not impair ILC2 function when extracellular Asn is sufficient. In contrast, under Gln-replete conditions, the combined absence of extracellular Asn and endogenous ASNS severely compromises ILC2 function and viability. These findings suggest that Gln supports ILC2 homeostasis primarily by sustaining intracellular Asn biosynthesis. Whether Gln supplementation alone can fully replenish intracellular Asn pools under conditions of Asn restriction remains an important question for future investigation. These findings suggest that Gln supports ILC2 homeostasis, at least in part, by sustaining intracellular Asn biosynthesis. We recognize that Gln, Glu, Asp, and Asn are metabolically interconnected. As discussed above, Gln may support ILC2 function not only by providing the amide nitrogen for ASNS-mediated Asn synthesis but also through glutaminolysis, glutamate production, anaplerotic fueling of the TCA cycle, and maintenance of intracellular Asp pools. The partial rescue by exogenous Asn under Gln-limited conditions suggests that Asn availability contributes to, but does not fully account for, the metabolic support provided by Gln. Future studies using intracellular metabolomics and isotope tracing will be necessary to define the relative contribution of ASNS-dependent and ASNS-independent Gln metabolism in ILC2s.

ASNS is a key metabolic enzyme that catalyzes the conversion of Asp and Gln into Asn and glutamate, thereby enabling *de novo* Asn biosynthesis to sustain intracellular Asn pools required for protein synthesis and cellular homeostasis. In CD8^+^ T cells, ASNS expression is minimal during early activation but becomes markedly upregulated after 24 hours, allowing cells to maintain cell survival independently of exogenous Asn (13). Consistent with this paradigm, our results show that ILC2-specific deletion of *Asns* has no detectable impact on intestinal ILC2s under steady-state conditions, whereas dietary Asn restriction significantly compromises their function. A limitation of the dietary Asn-deficiency model should also be acknowledged. Reduced luminal Asn availability may alter the intestinal microbiota, which could indirectly influence lamina propria immune cell populations. Gut commensal bacteria can utilize and modify intestinal amino acid pools, including Asn-related metabolic substrates, and microbiota-dependent changes in epithelial and mucosal immune signals may affect intestinal ILC2 responses. Therefore, the reduction in ILC2 frequency and IL-5 reporter activity observed under Asn-deficient dietary conditions should not be interpreted solely as a direct consequence of reduced Asn availability to ILC2s. Although our *in vitro* experiments using sorted LI ILC2s support a direct role for Asn availability and ASNS-dependent Asn biosynthesis in maintaining ILC2 survival and effector output, the *in vivo* dietary model may involve additional microbiota-mediated mechanisms. Future studies combining intestinal Asn quantification, microbiota profiling, fecal metabolomics, antibiotic or germ-free models, cohousing or fecal microbiota transplantation, and broader immune phenotyping will be necessary to clarify whether altered gut microbiota contribute to the effects of dietary Asn restriction on lamina propria immune cell populations.

Another related consideration is that dietary Asn restriction may not selectively affect ILC2s. Because Asn is a general nutrient required for protein synthesis and cellular homeostasis, fluctuations in environmental Asn availability may also influence other lamina propria immune cell populations, including T cells, B cells, eosinophils, macrophages, or other ILC subsets. In the present study, our *in vivo* analysis focused mainly on intestinal ILC2 frequency and IL-5 reporter activity; therefore, we cannot determine whether the observed changes are specific to ILC2s or occur as part of a broader remodeling of the intestinal immune landscape. Although our *in vitro* experiments using sorted LI ILC2s support a direct role for Asn availability in maintaining ILC2 survival and effector output, future studies incorporating broader immune phenotyping, single-cell transcriptomic analysis, and cell-type-specific functional assays will be required to define the relative sensitivity of different intestinal immune cell populations to environmental Asn restriction.

In addition to activation-dependent regulation, *Asns* expression in ILC2s is also highly responsive to extracellular nutrient availability. Under conditions of exogenous Asn deprivation, ILC2s markedly upregulate *Asns*, consistent with observations in B cells and CD8^+^ T cells (13–15). This adaptive response is likely mediated by the integrated stress response, in which amino acid limitation activates the transcription factor ATF4 (38). ATF4 directly induces *Asns* transcription, thereby promoting *de novo* Asn biosynthesis to compensate for reduced extracellular supply (39). Through this mechanism, ILC2s can partially buffer Asn scarcity and maintain cellular function and survival under nutrient-restricted conditions.

With respect to amino acid transport in ILC2 biology, previous studies have demonstrated that extracellular amino acid uptake mediated by SLC7A8 is essential for sustaining ILC2 effector function (8). However, the specific transporters responsible for Asn uptake and exchange in ILC2s remain unclear. To address this, we examined the expression of candidate Asn transporters previously characterized in CD8^+^ T cells (24). Our expanded transporter analysis suggests that ILC2 activation is accompanied by context-dependent remodeling of Asn/Gln transporter expression. For example, *Slc1a5, Slc7a5, Slc7a8, Slc38a1, and Slc38a2* display stimulus- and tissue-dependent changes. These findings indicate that Asn/Gln availability in activated ILC2s may be regulated by a broader transport network. However, because transcriptomic data do not directly measure transporter activity, substrate specificity, transport directionality, or intracellular amino acid flux, these observations should be interpreted as candidate pathway information rather than functional evidence of increased Asn or Gln uptake. Further transporter-specific perturbation, uptake assays, and isotope-tracing experiments will be needed to determine which transporters functionally regulate Asn/Gln availability in activated ILC2s. Although activated lung ILC2s displayed increased *Slc1a5* expression in public transcriptomic datasets, these data should be interpreted as supportive transcriptional observations. Because the present study focuses on intestinal ILC2s, further functional studies will be required to determine whether lung ILC2s rely on enhanced Asn uptake during type 2 airway inflammation.

## Data Availability

The raw data supporting the conclusions of this article will be made available by the authors, without undue reservation.
