# Supplementary material for: ASNS-dependent asparagine availability supports intestinal ILC2 responses and type 2 immunity under nutrient-limited conditions
Source: Front Immunol. 2026 Sep 15;17:1878920. doi: 10.3389/fimmu.2026.1878920 (PMC13619821; doi:10.3389/fimmu.2026.1878920)
Supplement: Supplementary file 1 [file DataSheet5.pdf]

Table S5 Antibodies used in this study

Antibodies	Source	Identifier
Anti-mouse CD16/CD32 (clone 93)	eBioscience	Cat# 14-0161-85
Anti-mouse CD45.2 Alexa Fluor 700 (clone 104)	eBioscience	Cat# 56-0454-82
Anti-mouse FcεRIα APC/Cyanine7 (clone MAR-1)	Biolegend	Cat# 134326
Anti-mouse CD11c APC/Cyanine7 (clone N418)	Biolegend	Cat# 117324
Anti-mouse CD5 APC-eFlour 780 (clone 53-7.3)	eBioscience	Cat# 47-0051-82
Anti-mouse TER-119 APC-eFlour 780 (clone TER-119)	eBioscience	Cat# 47-5921-82
Anti-mouse Ly-6G APC/Cyanine7 (clone 1A8)	Tonbo Biosciences	Cat# 25-1276
Anti-mouse/human CD45R/B220 APC/Cyanine7 (clone RA3-6B2)	Biolegend	Cat# 103224
Anti-mouse/human CD11b APC/Cyanine7 (clone M1/70)	Tonbo Biosciences	Cat# 25-0112
Anti-mouse CD16/32 APC/Cyanine7 (clone 93)	Bioligand	Cat# 101328
Anti-mouse CD3e APC-eFlour 780 (clone145-2C11)	eBioscience	Cat# 47-0031-82
Anti-mouse CD19 APC-eFlour 780 (clone 1D3)	eBioscience	Cat# 47-0193-82
Anti-mouse/human IL-5 APC (clone TRFK5)	Biolegend	Cat# 504306
Anti-mouse IL-13 Alexa Fluor 488 (clone eBio13A)	eBioscience	Cat# 53-7133-82
Anti-mouse GATA3 BV421 (clone A20)	BD Horizon	Cat# 563349
Anti-mouse KLRG1 PerCP/Cy5.5 (clone 2F1)	eBioscience	Cat# 46-5893-82
Anti-mouse CD127(IL-17Ra) APC (clone A7R34)	Biolegend	Cat# 135012
