# Supplementary material for: ASNS-dependent asparagine availability supports intestinal ILC2 responses and type 2 immunity under nutrient-limited conditions
Source: Front Immunol. 2026 Sep 15;17:1878920. doi: 10.3389/fimmu.2026.1878920 (PMC13619821; doi:10.3389/fimmu.2026.1878920)
Supplement: Supplementary file 2 [file DataSheet6.pdf]

Table S6 Composition of IMDM and DMEM culture media

	IMDM(CM10016)(mg/L)	DMEM(CM15019)(mg/L)	DMEM(-Gln)(CM15013)(mg/L)
**Inorganic salts**			
CaCl2(anhydrous)	165	200	200
Fe(NO3)3·9H2O	—	0.1	0.1
KCl	330	400	400
KNO3	0.076	—	—
MgSO4(anhydrous)	97.7	97.7	97.7
Na2SeO3	0.0173	—	—
NaCl	4505	6400	6400
NaH2PO4·H2O	125	125	125
NaHCO3	3024	3700	3700
**Amino acids**			
L-Alanine	25	—	—
L-Arginine·HCl	84	84	84
L-Asparagine·H2O	28.4	—	—
L-Aspartic acid	30	—	—
L-Cystine·2HCl	91.24	62.57	62.75
L-Glutamic acid	75	—	—
L-Glutamine	584	584	—
Glycine	30	30	30
L-Histidine·HCl·H2O	42	42	42
L-Isoleucine	105	104.8	104.8
L-Leucine	105	104.8	104.8
L-Lysine·HCl	146	146.2	146.2
L-Methionine	30	30	30
L-Phenylalanine	66	66	66
L-Proline	40	—	—
L-Serine	42	42	42
L-Threonine	95	95.2	95.2
L-Tryptophan	16	16	16
L-Tyrosine·2Na·2H2O	103.79	103.79	103.79
L-Valine	94	94	94
**Vitamins**			
Biotin	0.013	—	—
D-Calcium pantothenate	4	4	4
Choline chloride	4	4	4
Folic acid	4	4	4
i-Inositol	7.2	7.2	7.2
Nicotinamide	4	4	4
Para-Aminobenzoic acid	—	—	4
Pyridoxine·HCl	4	4	4
Riboflavin	0.4	0.4	0.4
Thiamine·HCl	4	4	4
Vitamin B12	0.013	—	—
**Other**			
D-Glucose	4500	4500	4500
Phenol red, Na	15	15	15
Sodium pyruvate	110	110	110
HEPES	5958	5958	—
