# Supplementary material for: ASNS-dependent asparagine availability supports intestinal ILC2 responses and type 2 immunity under nutrient-limited conditions
Source: Front Immunol. 2026 Sep 15;17:1878920. doi: 10.3389/fimmu.2026.1878920 (PMC13619821; doi:10.3389/fimmu.2026.1878920)
Supplement: Supplementary file 3 [file Image1.pdf]

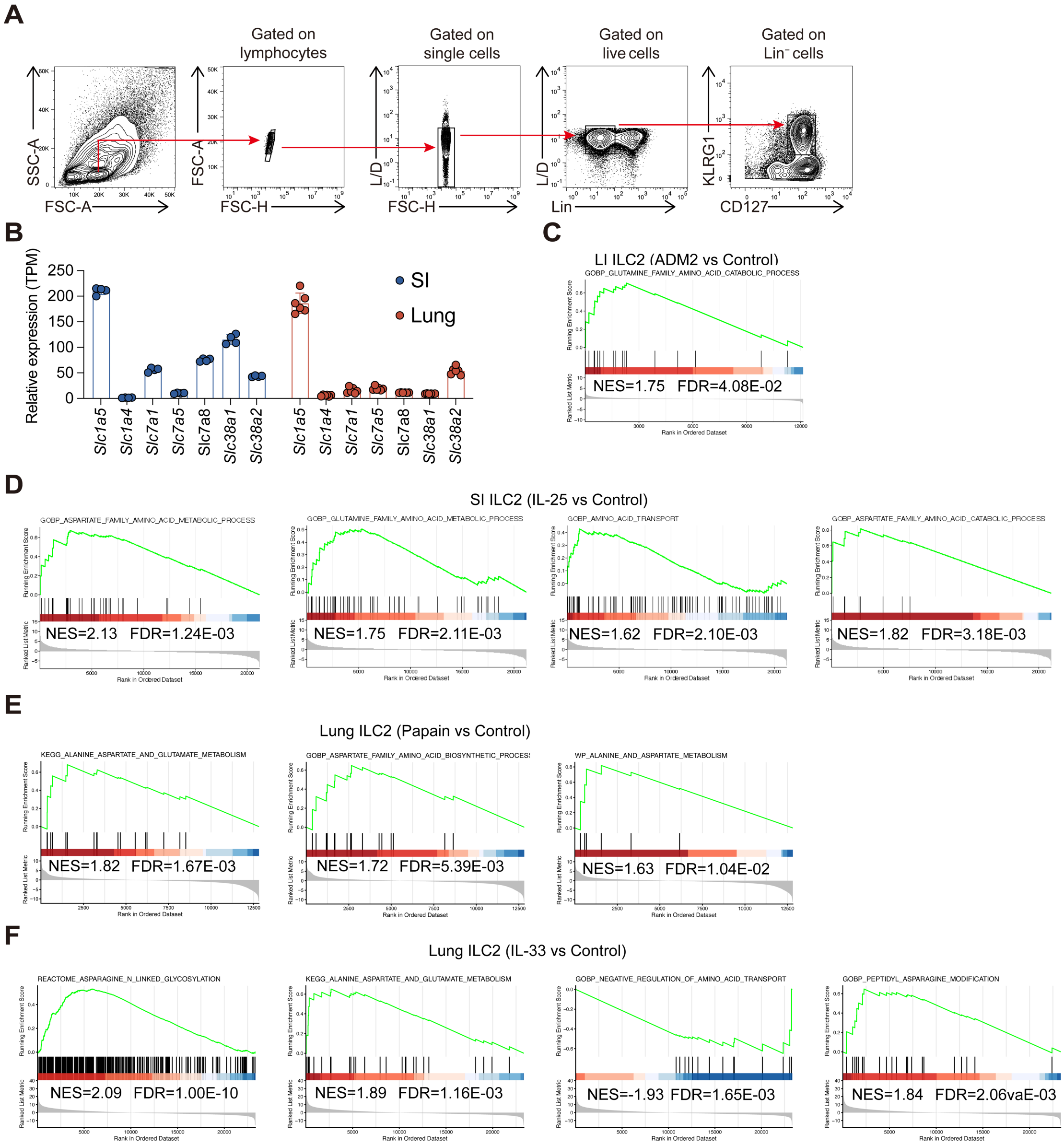

Supplementary Figure 1. Related to Figure 1. **(A)** For flow cytometry sorting, live cells were gated. Mouse ILC2 were gated base on: CD45.2$^+$ Lin$^-$ CD127$^+$ KLRG1$^+$. **(B)** Relative expression (TPM) of *Asns* transporter from naïve ILC2 isolated from small intestine (GSE205669) or lung (GSE148539). Each dot represents an individual mouse. **(C-F)** GSEA analysis related to dataset GSE271362 (C, related to Figure 1D), GSE205669 (D, related to Figure 1E), E-MTAB-15967 (E, related to Figure 1F) and GSE148539 (F, related to Figure 1G).

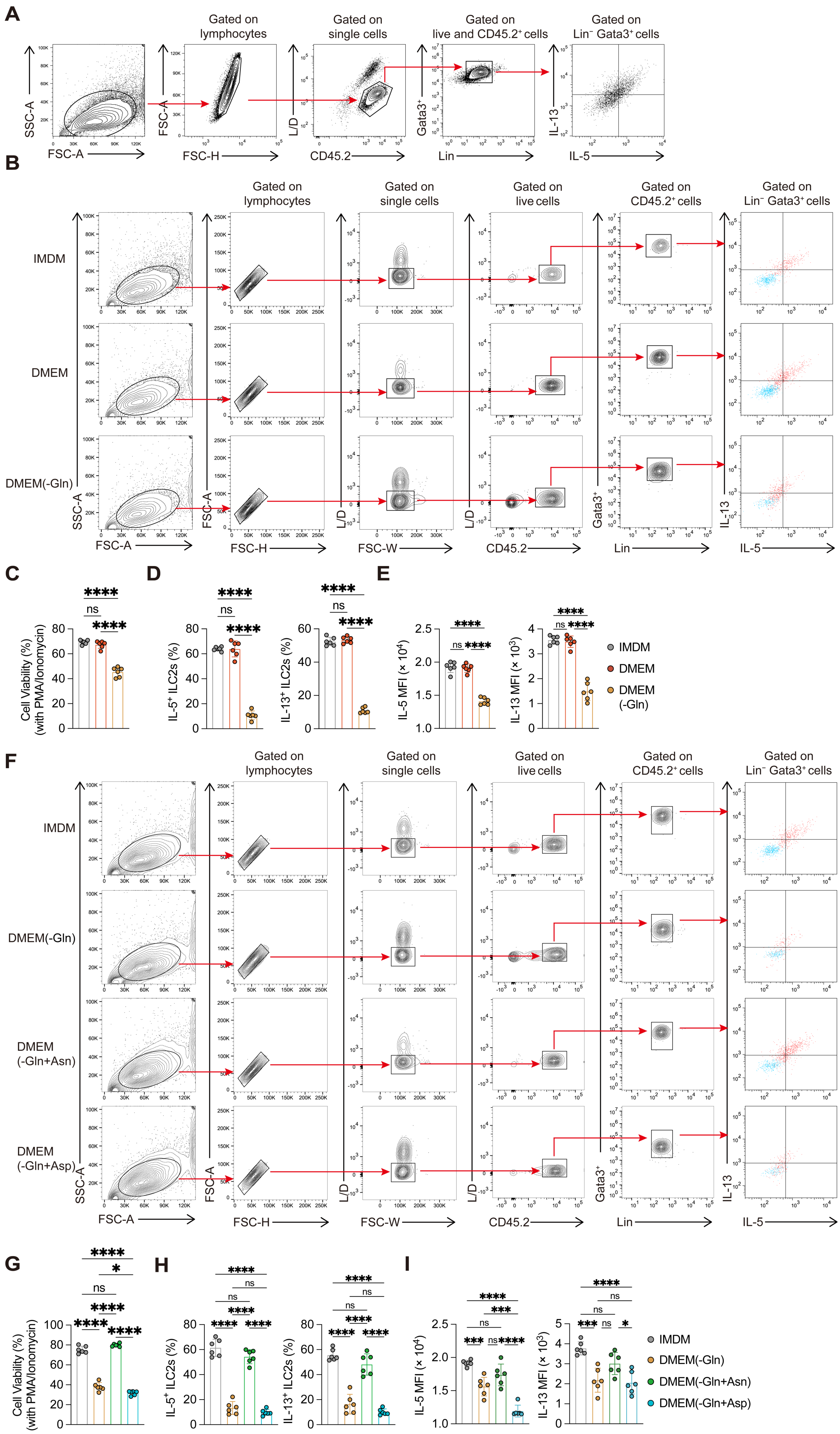

A
Gated on lymphocytes
Gated on single cells
Gated on live and CD45.2⁺ cells
Gated on Lin⁻ Gata3⁺ cells
SSC-A
FSC-A
FSC-A
FSC-H
L/D
CD45.2
Gata3⁺
Lin
IL-13
IL-5
B
IMDM
DMEM
DMEM(-Gln)
Gated on live cells
Gated on CD45.2⁺ cells
FSC-W
C
D
E
Cell Viability (%) (with PMA/Ionomycin)
IL-5⁺ ILC2s (%)
IL-13⁺ ILC2s (%)
IL-5 MFI (× 10⁴)
IL-13 MFI (× 10³)
ns
****
IMDM
DMEM
DMEM (-Gln)
F
DMEM (-Gln+Asn)
DMEM (-Gln+Asp)
G
H
I
*
***
DMEM(-Gln)
DMEM(-Gln+Asn)
DMEM(-Gln+Asp)

Supplementary Figure 2. Related to Figure 2. **(A)** For flow cytometry analysis, live cells were gated. Mouse ILC2 were gated base on: CD45.2^{+} Lin^{-} Gata3^{+}. **(B)** Gating strategy and fluorescence minus one (FMO) of ILC2s from indicated groups. Blue represents FMO, red represents IL-5/IL-13. **(C)** Quantification of the frequency of live cells (under PAM/Ionomycin/Brefeldin A stimulation, related to Figure 2D). **(D)** Quantification of the frequency of IL-5^{+} ILC2s and IL-13^{+} ILC2s (related to Figure 2E). **(E)** Quantification of MFI of IL-5 and IL-13 (related to Figure 2E). Each dot represents an individual mouse. **(F)** Gating strategy and FMO of ILC2s from indicated groups. Blue represents FMO, red represents IL-5/IL-13.**(G)** Quantification of the frequency of live cells (under PAM/Ionomycin/Brefeldin A stimulation, related to Figure 2K). Each dot represents an individual mouse. **(H)** Quantification of the frequency of IL-5^{+} ILC2s and IL-13^{+} ILC2s (related to Figure 2L). **(I)** Quantification of MFI of IL-5 and IL-13 (related to Figure 2L). Data are presented as mean ± SD. Statistical significance was determined by ordinary one-way ANOVA with Tukey's multiple comparisons test; ns, not significant; *$p < 0.05$; ***$p < 0.001$; ****$p < 0.0001$.

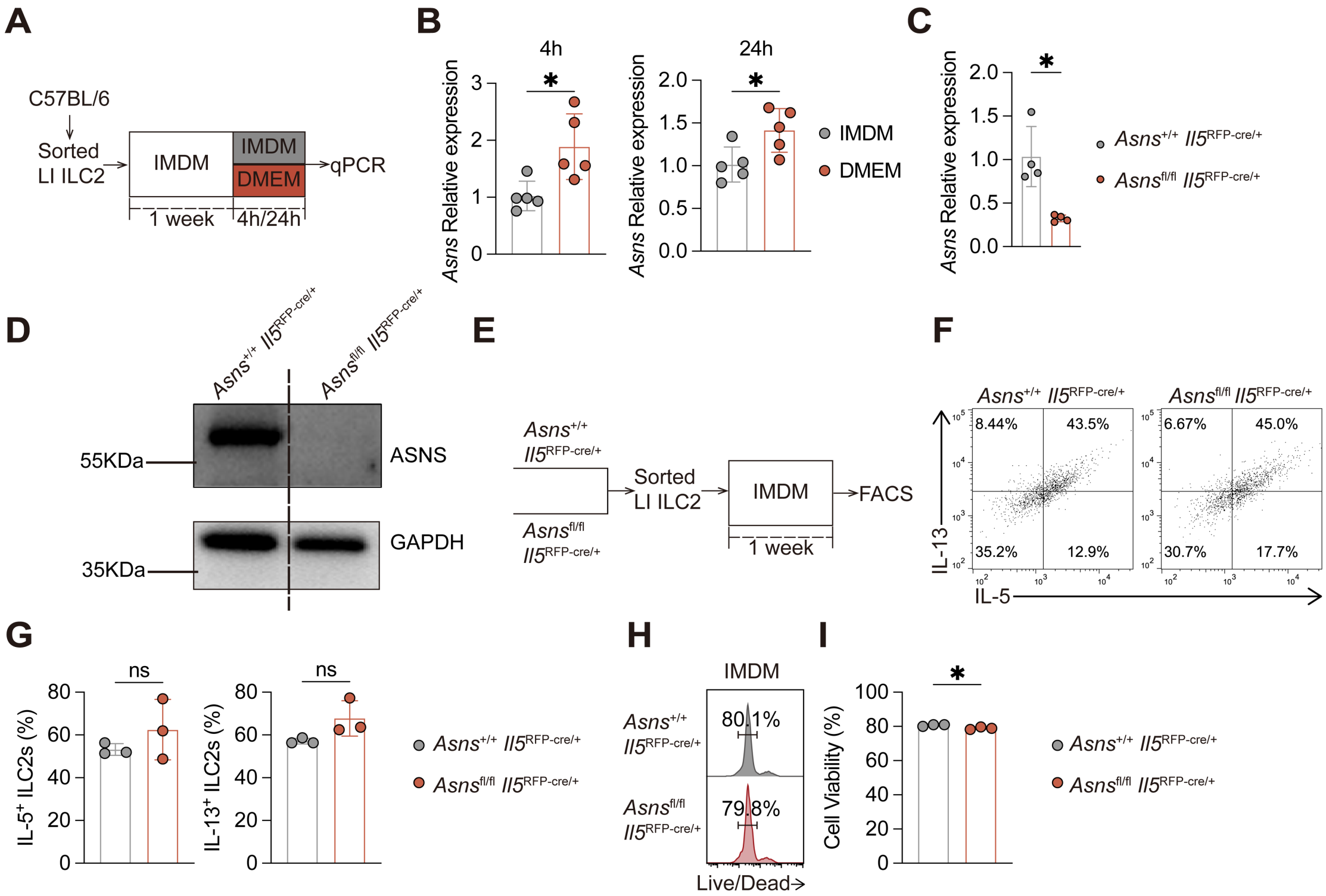

Supplementary Figure 3. Related to Figure 3. **(A-B)** LI lamina propria ILC2s were sorted from C57BL/6 mice, expanded in IMDM for 1 week, and then cultured in IMEM or DMEM for 4h or 24h, followed by qPCR analysis. Experimental workflow (A). *Asns* expression in indicated medium and indicated time (B). **(C-D)** Validation of Asns deficiency of isolated lamina propria ILC2s by qPCR (C) or Western blotting (D) analysis. **(E-I)** LI lamina propria ILC2s were sorted from *Asns*$^{+/+}$ *Il5*$^{\text{RFP-cre/+}}$ (control) or *Asns*$^{\text{fl/fl}}$ *Il5*$^{\text{RFP-cre/+}}$ (ILC2-specific *Asns*-deficient) mice and expanded in IMDM for 1 week, followed by flow cytometric analysis. Experimental workflow (E). Representative flow cytometry plots showing IL-5 and IL-13 production by ILC2s (F). Quantification of the frequency of IL-5^{+} ILC2s and IL-13^{+} ILC2s (G). Representative histograms showing the viability of ILC2s (H). Quantification of the frequency of live cells (I). Each dot represents an individual mouse. Data are presented as mean ± SD. Statistical significance was determined by Mann-Whitney test (B, C, G, I); ns, not significant; *$p < 0.05$.

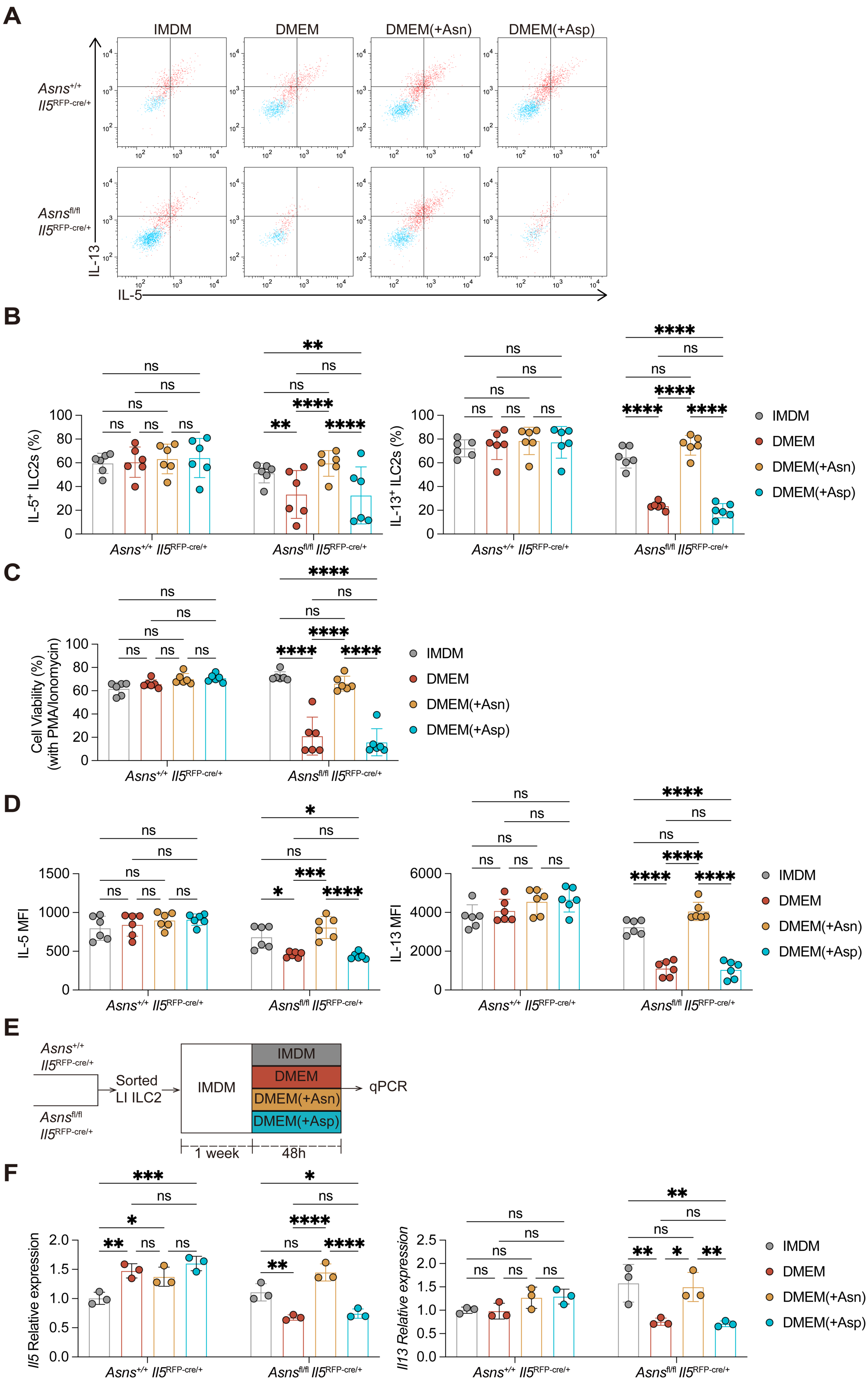

Supplementary Figure 4. Related to Figure 3. **(A)** FMO of ILC2s from indicated groups. Blue represents FMO, red represents IL-5/IL-13. **(B)** Quantification of the

frequency of IL-5^{+} ILC2s and IL-13^{+} ILC2s (related to Figure 3D). **(C)** Quantification of the frequency of live cells (under PAM/Ionomycin/Brefeldin A stimulation, related to Figure 3C). **(D)** Quantification of MFI of IL-5 and IL-13 (related to Figure 3D). **(E, F)** LI lamina propria ILC2s were sorted from *Asns*$^{+/+}$ *Il5*$^{\text{RFP-cre/+}}$ (control) or *Asns*$^{\text{fl/fl}}$ *Il5*$^{\text{RFP-cre/+}}$ (ILC2-specific *Asns*-deficient) mice and expanded in IMDM for 1 week. Cells were then cultured for 48 h in IMDM, DMEM and DMEM supplemented with Asn (28.4 mg/L, DMEM(+Asn)) or Asp (30 mg/L, DMEM(+Asp)), followed by qPCR analysis. Experimental workflow (D). *Il5* and *Il13* expression in indicated time (E). Each dot represents an individual mouse. Data are presented as mean ± SD. Statistical significance was determined by ordinary two-way ANOVA with Tukey's multiple comparisons test; ns, not significant; *p < 0.05; **p < 0.01; ***p < 0.001; ****p < 0.0001.

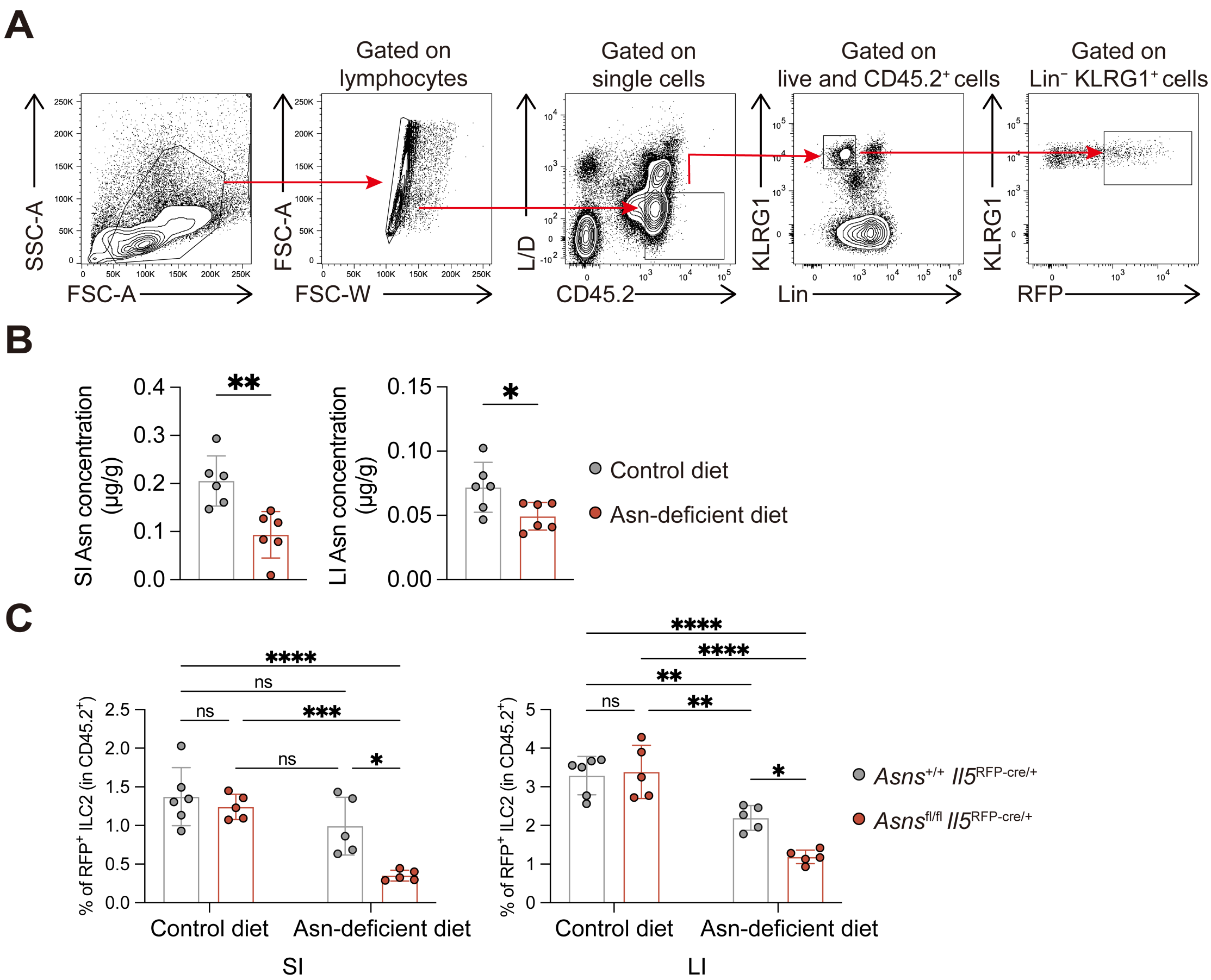

Supplementary Figure 5. Related to Figure 4. **(A)** Gating strategy of intestinal lamina propria ILC2s by flow cytometry. For flow cytometry analysis, live cells were gated. Mouse ILC2 were gated base on: $CD45.2^+$ Lin^- $KLRG1^+$. **(B)** HPLC-MS analysis of mouse SI and LI Asn concentration under control diet or Asn-deficient diet. **(C)** Quantification of the frequency of $KLRG1^+$ ILC2s in all $CD45.2^+$ cells from the SI and LI lamina propria lymphoid. Data are presented as mean ± SD. Statistical significance was determined by unpaired t-test (B) or by ordinary two-way ANOVA with Tukey's multiple comparisons test (C); ns, not significant; *$p < 0.05$; **$p < 0.01$; ***$p < 0.001$; ****$p < 0.0001$.
